# Evaluation of patients with relapsing-remitting multiple sclerosis using tract-based spatial statistics analysis: diffusion kurtosis imaging

**DOI:** 10.1186/s12883-018-1108-2

**Published:** 2018-08-07

**Authors:** Hai Qing Li, Bo Yin, Chao Quan, Dao Ying Geng, Hai Yu, Yi Fang Bao, Jun Liu, Yu Xin Li

**Affiliations:** 1 Department of Radiology, Huashan Hospital, Fudan University, 12 Wulumuqi Rd. Middle, Shanghai, 200040 China; 2 Department of Neurology, Huashan Hospital, Fudan University, Shanghai, China; 3 Institute of Functional and Molecular Medical Imaging, Fudan University, Shanghai, China; 4 Department of Radiology, The Fifth People’s Hospital of Shanghai, Fudan University, 128 Ruili Rd, Shanghai, 200240 China

**Keywords:** Multiple sclerosis, relapsing–remitting, Diffusion kurtosis imaging, Diffusion tensor imaging, Tract-based spatial statistics

## Abstract

**Background:**

Diffusion kurtosis imaging (DKI) has the potential to provide microstructural insights into myelin and axonal pathology with additional kurtosis parameters. To our knowledge, few studies are available in the current literature using DKI by tract-based spatial statistics (TBSS) analysis in patients with multiple sclerosis (MS). The aim of this study is to assess the performance of commonly used parameters derived from DKI and diffusion tensor imaging (DTI) in detecting microstructural changes and associated pathology in relapsing remitting MS (RRMS).

**Methods:**

Thirty-six patients with RRMS and 49 age and sex matched healthy controls underwent DKI. The brain tissue integrity was assessed by fractional anisotropy (FA), mean diffusivity (MD), axial diffusivity (Da), radial diffusivity (Dr), mean kurtosis (MK), axial kurtosis (Ka) and radial kurtosis (Kr) of DKI and FA, MD, Da and Dr of DTI. Group differences in these parameters were compared using TBSS (*P* < 0.01, corrected). To compare the sensitivity of these parameters in detecting white matter (WM) damage, the percentage of the abnormal voxels based on TBSS analysis, relative to the whole skeleton voxels for each parameter was calculated.

**Results:**

The sensitivities in detecting WM abnormality in RRMS were MK (78.2%) > Kr (76.7%) > Ka (53.5%) and Dr (78.8%) > MD (76.7%) > FA (74.1%) > Da (28.3%) for DKI, and Dr (79.8%) > MD (79.5%) > FA (68.6%) > Da (40.1%) for DTI. DKI-derived diffusion parameters (FA, MD, and Dr) were sensitive for detecting abnormality in WM regions with coherent fiber arrangement; however, the kurtosis parameters (MK and Kr) were sensitive to discern abnormalities in WM regions with complex fiber arrangement.

**Conclusions:**

The diffusion and kurtosis parameters could provide complementary information for revealing brain microstructural damage in RRMS. Dr and DKI_Kr may be regarded as useful surrogate markers for reflecting pathological changes in RRMS.

## Background

Multiple sclerosis (MS) is a chronic disorder of the CNS, characterized by focal white matter (WM) plaques along with diffuse normal appearing WM (NAWM) damage and cortical demyelination [1]. Diffusion tensor imaging (DTI) is one of the most widely used methods in detecting microstructural abnormalities based on water diffusion measures with the assumption that the diffusion displacement of water molecule in an unrestricted environment has a Gaussian approximation [2].In reality, water molecules often show non-Gaussian diffusion due to the presence of barriers of cell membranes, axon sheaths, and water compartments in biological tissues [3]. So it is thought that DTI may not be capable to provide accurate values at dense intersections of fiber tracts [4]. In contrast, as a clinically feasible extension of DTI, diffusion kurtosis imaging (DKI) has been proposed to characterize the deviation of water diffusion in neural tissues from Gaussian diffusion [5, 6]. Both diffusion parameters including fractional anisotropy (FA), mean diffusivity (MD), axial diffusivity (Da), radial diffusivity (Dr) and kurtosis parameters including mean kurtosis (MK), axial kurtosis (Ka), radial kurtosis (Kr) could be obtained from DKI data. DKI can be regarded as a more sensitive indicator of diffusional heterogeneity and can be used to investigate abnormalities in tissues with isotropic structure [6, 7].

The sensitivity of DKI has been evaluated in age-related diffusion patterns in the prefrontal brain [8], reactive astrogliosis in traumatic brain injury [9], and cuprizone-induced demyelination in mice [10], which showed better demonstration of microstructural changes than with DTI. However, there were few studies to validate the merits of DKI in evaluating patients with MS [11, 12]. Tract-based spatial statistics (TBSS) provides a powerful and objective method to perform multi-subject comparisons [13].

In this study, the microstructural alterations reflected by both DKI and DTI parameters in relapsing-remitting multiple sclerosis (RRMS) were investigated using TBSS. Our aim is to assess the performances of 11 commonly used parameters derived from DKI (MK, Ka, Kr, FA, MD, Da and Dr) and DTI (FA, MD, Da and Dr) in detecting microstructural abnormalities in RRMS.

## Methods

### Subjects

Thirty-six (13 male and 23 female) consecutive patients with RRMS (diagnosed by McDonald criteria [14]) were prospectively enrolled in this study. All patients underwent clinical assessments, including relapse history and disability assessment using EDSS before MRI examination. Patients were excluded if they had a history of other CNS disorders, corticosteroid use or relapses within three months prior to MRI. For comparison, 49 age- and gender-matched healthy controls (17 male and 32 female), with no previous history of neurological disorders were recruited (Table 1). Approval for this study was obtained from the Ethics Committee of Huashan Hospital, Fudan University and written informed consent was obtained from all the subjects.

**Table 1:** Demographic and clinical characteristics of RRMS patients and healthy controls

Characteristics	RRMS patients	Healthy controls	*P* value
Number of subjects	36	49	
Age (years)	32.9 ± 10.6	32.3 ± 10.6	0.8*
Sex (male: female)	13:23	17:32	0.9^★^
EDSS	1.5 (0–5)^a^	NA	
Disease duration(month)	54.5 ± 62.8	NA	
White matter lesion volume (ml)	17.94 ± 19.00	NA	

^*^A Chi-square test of Pearson and ^★^a *t*-test of Student were used to test the group differences in sex and age respectively. The data were shown as the mean values ± standard deviations. Abbreviations: *EDSS* expanded disability status scale, ^a^median is 1.5, interquartile range is 1,1.5,2.375, minimum-maximum is 0–5. *NA* not applicable

### MRI data acquisition

All scans were acquired using Discovery MR750 3.0 T scanner (GE Healthcare, Milwaukee, WI, USA) with an eight-channel phase array head coil.

An axial FLAIR sequence (SE: repetition time/ echo time = 8800/146 ms, slice thickness = 6.0 mm, field of view =512 × 512 mm, voxel size = 0.5 × 0.5 × 6.0 mm^3^) for white matter lesion volume calculation was performed. DKI was acquired with two values of b (b = 1250 and 2500 s/mm^2^) along 25 diffusion-encoding directions and b value of 0 s/mm^2^ along 25 non-diffusion-weighted images, with a spin-echo single-shot echo planar imaging (EPI) sequence (TR/TE = 4700/102 ms; matrix = 128 × 128; FOV = 240 × 240 mm; slice thickness = 4 mm without gap; 35 axial slices; acquisition time was 8 min and 42 s).

### White matter lesion volume calculation

With MRIcron software (http://www.nitrc.org/projects/mricron/) we drew all the WM leisons manually on FLAIR images and calculated total WM lesion volume for each patient and summerized it in Table 1.

### DKI data processing

We used the same methodology from a previously published work [15], and the differences were as following. In “calculation of diffusion and kurtosis parameters”, all the data (b = 0, 1250, 2500 s/mm^2^) were used for DKI fitting and only images with b = 0 and 1250 s/mm^2^ were employed for DTI fitting. In “tract-based spatial statistics”, group comparisons between RRMS patients and healthy controls were performed using a general linear model. The percentage of the abnormal voxels relative to the whole skeleton voxels for each parameter was calculated, so as to quantitatively compare the sensitivity of parameters from DKI and DTI in detecting brain tissue integrity impairments in RRMS.

## Results

### Kurtosis parameters from DKI

**Fig. 1:**
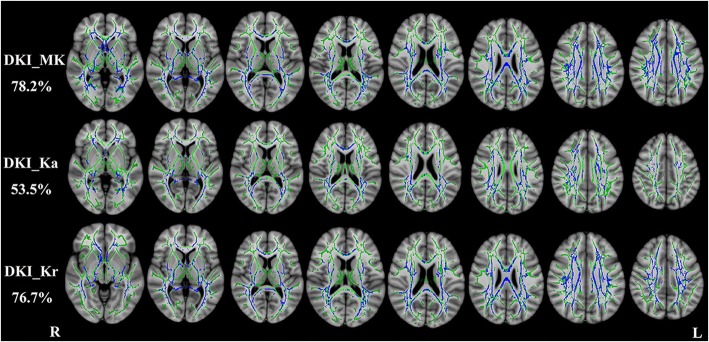
TBSS shows WM regions with significant differences in the DKI_MK, DKI_Ka and DKI_Kr between RRMS patients and healthy subjects (*P* < 0.01, FWE corrected). Green represents mean FA skeleton of all participants; blue represents reduction in RRMS patients. The percentage in the left column represents the percentage of the abnormal voxels relative to the whole skeleton voxels for each parameter

Compared with healthy controls, RRMS patients had significantly decreased DKI-derived kurtosis parameters in WM regions (*P* < 0.01, two-tailed, FWE corrected) with complex fiber arrangement, such as in the juxtacortical WM and corona radiata. DKI_MK, DKI_Ka and DKI_Kr could detect abnormal diffusion in 78.2%, 53.5% and 76.7% voxels of the whole WM skeleton respectively. Kurtosis parameters are shown in Fig. 1.

### Diffusion parameters from DKI

**Fig. 2:**
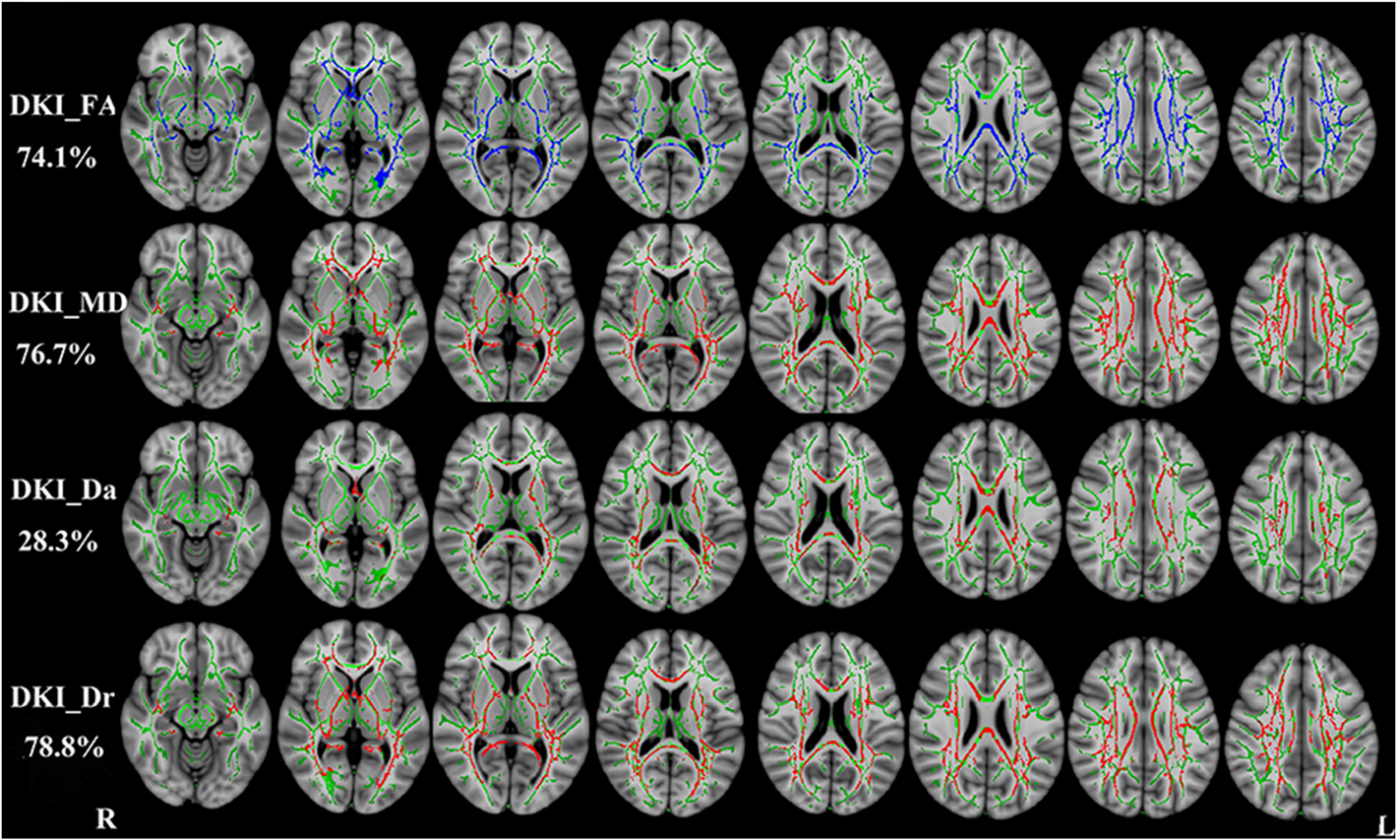
TBSS shows WM regions with significant differences in the DKI_FA, DKI_MD, DKI_Da and DKI_Dr between RRMS patients and healthy subjects (*P* < 0.01, FWE corrected). Green represents mean FA skeleton of all participants; red denotes increase and blue represents reduction in RRMS patients. The percentage in the left column represents the percentage of the abnormal voxels relative to the whole skeleton voxels for each parameter

Compared with healthy controls, RRMS patients demonstrated reduced DKI_FA in WM regions with coherent fiber arrangement, such as the corpus callosum and anterior limb of internal capsule, and increased DKI_MD, DKI_Da and DKI_Dr (*P* < 0.01, two-tailed, FWE corrected). DKI_FA, DKI_MD, DKI_Da and DKI_Dr could detect abnormal diffusion in 74.1%, 76.7%, 28.3% and 78.8% voxels of the whole WM skeleton respectively. DKI-derived diffusion parameters are shown in Fig. 2.

### Diffusion parameters from DTI

**Fig. 3:**
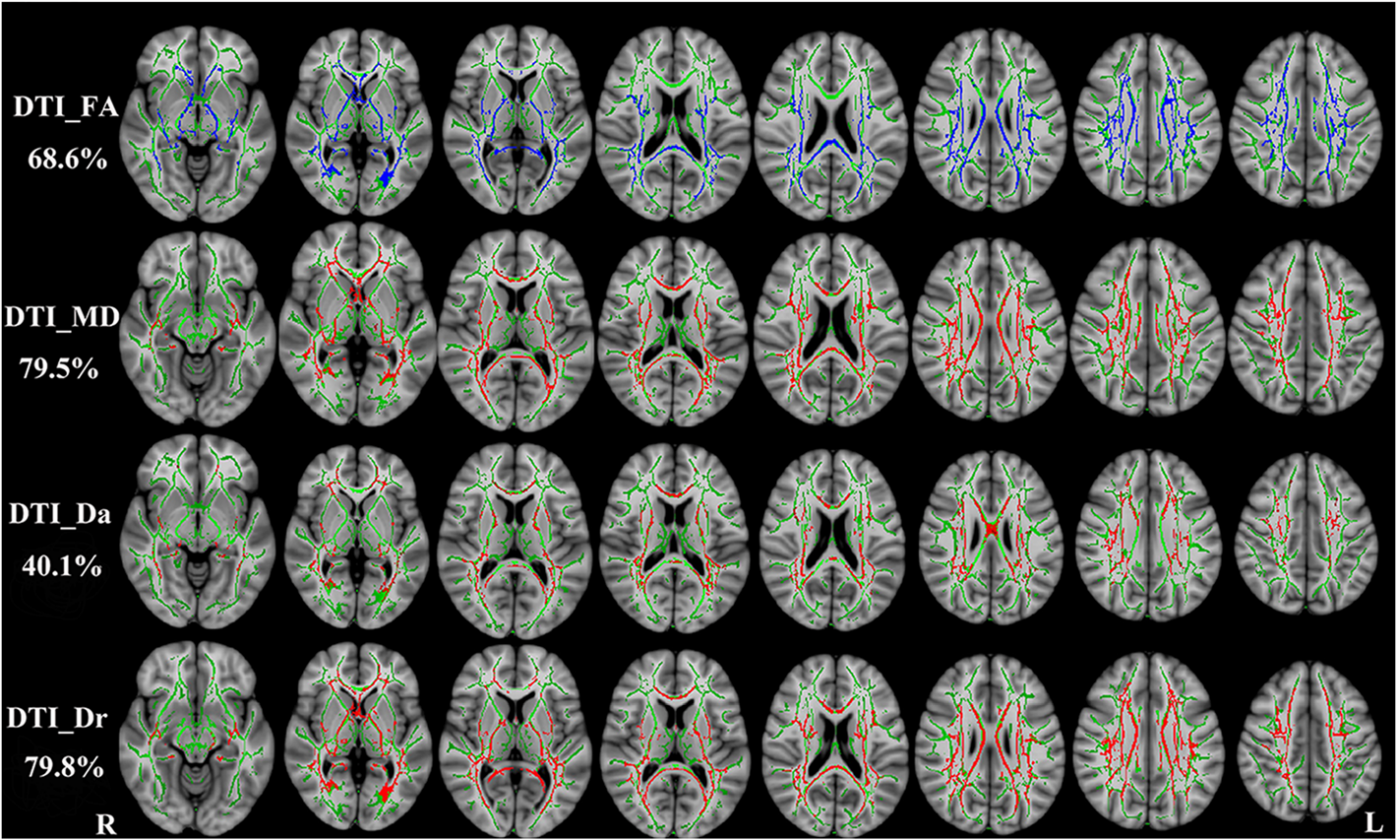
TBSS shows WM regions with significant differences in the DTI_FA, DTI_MD, DTI_Da and DTI_Dr between RRMS patients and healthy subjects (*P* < 0.01, FWE corrected). Green represents mean FA skeleton of all participants; red denotes increase and blue represents reduction in RRMS patients. The percentage in the left column represents the percentage of the abnormal voxels relative to the whole skeleton voxels for each parameter

RRMS patients exhibited similar patterns with DKI-derived diffusion parameters. FA was reduced, MD, Da and Dr were increased (*P* < 0.01, two-tailed, FWE corrected). DTI_FA, DTI_MD, DTI_Da and DTI_Dr could detect abnormal diffusion in 68.6%, 79.5%, 40.1% and 79.8% voxels of the whole WM skeleton respectively. DTI-derived diffusion parameters are shown in Fig. 3.

## Discussion

Although DTI has been widely used in investigating structural changes in the NAWM in MS [16, 17], it may not provide accurate parameters at dense intersections of fiber tracts [4]. In contrast, DKI can be used to quantify non-Gaussian diffusion, thus providing accurate parameters at dense intersection of fiber tracts [6]. To our knowledge, there were only a limited number of studies using DKI in MS patients [11, 12, 18, 19], Raz E et al. measured FA, MD, and MK values of the entire cross-sectional cord area, normal-appearing gray matter (NAGM) and WM in MS patients by DKI using region-of-interest (ROI) analysis, they thought that DKI could provide additional and complementary information to DTI on spinal cord pathology [11]. In another research, DKI was used to evaluate diffusional changes in NAWM regions remote from MS plaques using ROI analysis, the results indicated that DKI might be an additional sensitive indicator for detecting tissue damage in MS patients [12]. They concluded that DKI was sensitive for detecting tissue damage in MS patients and could provide information that was complementary to that of conventional DTI-derived metrics. However, most of these above-mentioned studies adopted ROI-based analysis, which had poor reproducibility of ROI positioning and only a limited number of specific regions can be examined. In contrast, the TBSS method used in this study was relatively a novel hypothesis-free and user-independent voxel-wise analysis.

In this study, TBSS analysis of both DKI and DTI derived parameters showed widespread WM damage in RRMS patients compared with healthy controls, which was consistant with previous studies using DKI [19] or DTI [20, 21]. Similarly to a research study using DKI in schizophrenia patients, we also observed that DKI-derived kurtosis and diffusion parameters had differernt sensitivity to detect abnormality in WM areas with different fiber architecture [15]. Moreover, we found that the MK decrease in the WM of RRMS patients was predominantly caused by the Kr decrease, and the FA decrease was mainly driven by the increase of Dr.

FA measures anisotropic water diffusion and is proven to be most applicable for assessing WM regions with coherent fiber arrangement. However, it is not suitable for detecting diffusion changes of complex WM architecture, such as crossing fiber regions [22, 23]. As the most characteristic parameter of DKI, MK measures the deviation of the diffusion displacement profile from a Gaussian distribution and enables to probe WM regions with complex fiber arrangement [24]. Therefore, the combination of diffusion and kurtosis parameters may provide improved sensitivity and specificity in detecting alterations in various WM structures. This theoretical prediction has been validated by a previous study in schizophrenia patients [15], and confirmed by our findings that altered diffusion parameters (especially reduced DKI_FA) were observed mainly in WM regions with coherent fiber arrangement (such as the corpus callosum and anterior limb of internal capsule). The percentage of abnormal DKI_FA voxels (74.1%) relative to the whole skeleton voxels was higher than that of DTI_FA (68.6%) in this study, which suggest that DKI_FA might have higher sensitivity than DTI_FA in detecting abnormality in WM regions, while reduced kurtosis parameters were mainly located in WM regions with complex fiber arrangement (such as the juxtacortical WM and corona radiate). The percentage of abnormal MK voxels relative to the whole skeleton voxels was 78.2%, which suggest that MK might have higher sensitivity than DTI in detecting abnormality in WM regions. Therefore, appropriate DKI derived parameters should be selected to probe altered diffusion pattern in specific WM regions in RRMS patients.

As we know, there is strong directional dependence of water distribution within myelinated WM tracts. However, once inflammation and demyelination occur, diffusivity will increase and directionality will decrease. The increase of diffusivity manifested as increase of Dr (diffusion perpendicular to the long axis) and Da (diffusion along the long axis). However, decreased Da was reported in some animal experiments [25, 26]. In our opinion, these studies may not take into account the full complexity of pathological processes occurred in RRMS. The increase of Da found in our RRMS patients was consistent with a TBSS study using DTI in RRMS patients [20]. The cause may be explained by severe decreases in axonal packing density which would lead to a whole increase in extracellular water, resulting in larger Dr increases and subsequent Da increases. Other reported reasons include fiber re-organization, increased axonal diameter and membrane permeability [27, 28]. In our study, the percentage of abnormal DKI_Dr voxels (79.8%) relative to the whole skeleton voxels is significantly higher than that of DKI_Da (28.3%), which demonstrated that the increase of MD (mean diffusivity) and decreased FA mainly caused by the increased DKI_Dr. Similarly, this pattern of changes was also found in DTI_Da and DTI_Dr. Interestingly, when assessing the contribution of DKI_Ka and DKI_Kr in those regions showing significantly decreased DKI_MK, we found these were driven predominantly by decreases in DKI_Kr (76.5% vs DKI_Ka 53.5%). All these above-mentioned findings suggested demyelination might be regarded as a key factor among various pathological changes in RRMS. So Dr and DKI_Kr might be regarded as useful surrogate markers for reflecting pathological changes and improving clinical–radiological correlations in MS. Furthermore, Dr and DKI_Kr measured by TBSS might have great potential to be a MRI biomarker in monitoring remyelination in MS patients.

## Conclusions

In conclusion, DKI-derived parameters were sensitive to detect abnormality in microstructural changes. The diffusion and kurtosis parameters could provide complementary information for revealing pathological changes in RRMS patients. Dr and DKI_Kr might be regarded as a useful surrogate marker for reflecting pathological changes and improving clinical–radiological correlations in MS patients.

## Abbreviations

Da: Axial diffusivity
Dr: Radial diffusivity
GM: Gray matter
Ka: Axial kurtosis
Kr: Radial kurtosis
MK: Mean kurtosis
NAGM: Normal-appearing gray matter
NAWM: Normal-appearing white matter
WM: White matter
